# Global trends and hotspots in research of robotic surgery in oncology: A bibliometric and visual analysis from 2002 to 2021

**DOI:** 10.3389/fonc.2022.1055118

**Published:** 2022-11-11

**Authors:** Hua Lu, Tingliang Han, Fangcun Li, Jiali Yang, Zhaomeng Hou

**Affiliations:** ^1^ Department of Orthopedics and Traumatology, Yancheng TCM Hospital Affiliated to Nanjing University of Chinese Medicine, Yancheng, China; ^2^ Department of Rehabilitation Medicine, Guilin Municipal Hospital of Traditional Chinese Medicine, Guilin, China; ^3^ Faculty of Orthopedics and Traumatology, Guangxi University of Chinese Medicine, Nanning, China

**Keywords:** robotic surgery, oncology, VOSviewer, CiteSpace, bibliometric, visual analysis

## Abstract

**Background:**

With the development of robotic surgery in the field of oncology, an increasing number of relevant research papers have been published. In order to explore the research hotspots and trends in this field, a bibliometric and visual analysis was performed for the first time.

**Methods:**

The literature records related to oncology robotic surgery were obtained from the Web of Science Core Collection database and imported into the software VOSviewer 1.6.18, CiteSpace 6.1.R3, and the Bibliometric Online Analysis Platform for analysis.

**Results:**

A total of 6,964 publications, including 5,635 articles and 1,329 reviews, were included in this study. Over the past 20 years, annual publications and citations have experienced rapid growth, particularly in the last two years. The United States was the country with the most publications, while Yonsei University in South Korea was the most productive institution. The *Journal of Robotic Surgery* and the *Journal of Urology* were the journals with the most publications and citations, respectively. Mottrie A from Belgium and Ficarra V from Italy were the authors with the highest number of publications and citations, respectively. The keywords “robotic surgical procedure”, “laparoscopic surgery”, “prostate cancer”, “colorectal cancer”, “gastric cancer”, “resection”, “complications classification”, “open surgery”, “transoral robotic surgery”, “pathological outcomes”, and “robot-assisted surgery” reflect the research hotspots and trends of oncology robotic surgery.

**Conclusion:**

The therapeutic advantages of robotic surgery in oncology are not yet prominent, and further randomized controlled trials with multicenter and large samples are needed to evaluate the advantages of robotic surgery compared with laparoscopic surgery and open surgery in the treatment of tumors from multiple outcome indicators.

## Introduction

Robotic surgery has experienced rapid development in the past 20 years and has received more and more attention around the world. It has been widely used in many surgical fields (1–4). The robotic surgical system provides surgeons with 3D high-definition surgical vision, multi-joint instruments, tremor filtering, and motion scaling, thereby improving the accuracy of complex anatomy and the flexibility of operation. It can also perform many surgical operations with minimal invasion (5–12). Compared with traditional open surgery, robotic surgery has less bleeding during the operation and shorter hospitalization time after the operation due to its advantages of minimally invasive technology, which not only improves the quality of life of patients but also reduces the occurrence of surgical complications (13–15). Furthermore, many studies in different fields of oncology surgery have shown the safety and feasibility of the robotic platform, as well as comparable results with conventional laparoscopy (16, 17). Nowadays, robotic surgery has been widely used in colorectal neoplasms, urologic neoplasms, gynecological tumors, etc. (18–20). However, with the development of robotic surgery, more and more related studies in the field of oncology have been published, but related bibliometric research has not been reported.

Bibliometrics research aims to explore the key paths and knowledge turning points in the evolution of a discipline through the measurement of literature in a specific field, and to analyze the potential dynamic mechanisms of discipline evolution and detect the frontiers of discipline development through drawing a series of visual maps (21, 22). Because the structure, regularity, and distribution of scientific knowledge are presented by means of visualization, the visual graphs obtained through such methods are called “scientific knowledge maps”. The so-called knowledge map is a kind of graph that takes the knowledge domain as an object to show the relationship between the development process and the structure of scientific knowledge. It has the dual properties and characteristics of “graph” and “spectrum”: it is not only a visual knowledge graph, but also a serialized knowledge genealogy, showing many implicit complex relationships between knowledge units or knowledge groups, such as network, structure, interaction, crossover, evolution, or derivation, and these complex knowledge relationships are breeding new knowledge generation (23). At present, a variety of visual analysis software has been developed and applied. VOSviewer, developed by Nees Jan van Eck and Ludo Waltman from Leiden University in the Netherlands, and CiteSpace, developed by Chaomei Chen from Drexel University in the United States, are the two most widely used visual analysis software, which have been widely used in many research fields (24, 25).

Our study visually analyzed the literature of robotic surgery in oncology research in the past 20 years included in the Web of Science Core Collection (WoSCC) database by using the software CiteSpace 6.1.R3, VOSviewer 1.6.18, and the Bibliometric Online Analysis Platform (https://bibliometric.com/). By drawing scientific knowledge maps, the research status, hotspots, and trends in this research field are presented in an intuitive manner, providing valuable references for further research.

## Materials and methods

### Data source and retrieval strategy

The WoSCC database was used as the data source, and all literature retrieval and data extraction were completed on September 1, 2022, in order to avoid the deviation caused by database updates. To improve the retrieval accuracy, the subject entries were obtained from the standardized Medical Subject Headings (MeSH) list of the National Library of Medicine. The combination of subject headings and free words was used for retrieval, and the retrieval strategy was: ((((TS = (robotic surgical procedure OR robotic assisted surgery OR robot surgery OR robot enhanced procedure)) AND TS = (neoplasm OR neoplasia OR cancer OR tumor)) AND DT = (Article OR Review)) AND LA = (English)) AND DOP = (2002-01-01/2021-12-31).

### Bibliometric analysis

The publications retrieved from the WoSCC database were exported in a plain text file with “full record and cited references” as the record content and named as “download_xxx.txt”, and then the downloaded documents were imported into CiteSpace 6.1.R3 software. No duplicate documents were found. At the same time, all documents were exported in UTF-8 format with “full record and cited references” and imported into the Bibliometric Online Analysis Platform to analyze cooperation between countries/regions. CiteSpace was used for the analysis of keyword bursts, keyword timeline maps, the dual-map overlay of journals, and co-cited reference bursts. VOSviewer was used to analyze author, institution, and country collaboration networks; keyword co-occurrence networks; and reference, author, and journal co-citation networks. CiteSpace software parameter settings: Time Span: January 2002 to December 2021; Years Per Slice: 2; Node Types: keyword, reference; Selection Criteria: top 50 per slice; Pruning: pathfinder, pruning sliced networks, and pruning the merged network. The remaining settings maintain the software default. VOSviewer software parameter settings: Normalization Method: association strength; the minimum publication thresholds for countries/regions, institutions, and authors are 5, 40, and 25, respectively; the minimum citation thresholds for journals, authors, and references are 600, 200, and 120, respectively; and the minimum keyword occurrence threshold is 100.

## Results and discussion

### Annual publications

**Figure 1:**
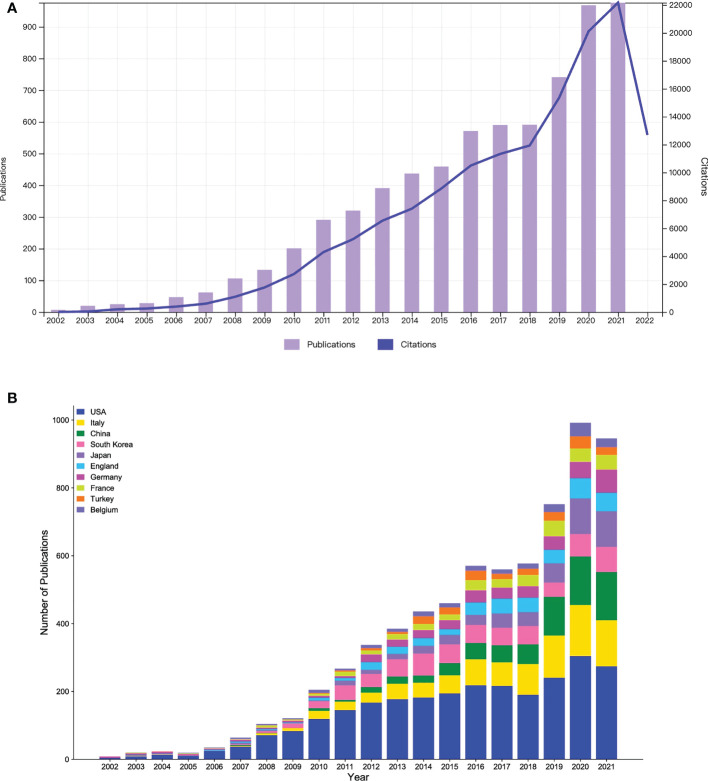
**(A)** The number of annual publications and citations on robotic surgery in oncology from 2002 to 2021. **(B)** The number of annual publications in oncology robotic surgery in the top 10 countries/regions between 2002 and 2021.

Excluding other types of publications such as meeting abstracts, editorial materials, letters, corrections, book chapters, and retracted publications, a total of 6,964 publications, including 5,635 articles and 1,329 reviews, were included in this study. **Figure 1A** shows the trend change in the number of annual publications and citations from 2002 to 2021. The results show that in the past 20 years, the research of robotic surgery in oncology has achieved rapid development and has received wide attention from many scholars. Especially in the last two years, the surge in the number of publications and citations indicates the high enthusiasm of researchers in this field. **Figure 1B** shows the number and growth trend of annual publications in the top 10 countries/regions in total publications over the past 20 years. It can be found that the United States was a major contributor to this research field, with the largest and steadily growing number of publications. Research progress in Italy and China was slow from 2002 to 2011, and research in China started late, with only two papers were published until 2007. However, in the past 10 years, the number of annual publications in the two countries has grown rapidly, and now they have become the top three countries in terms of the number of publications, second only to the United States.

### Distribution of countries/regions and institutions

**Figure 2:**
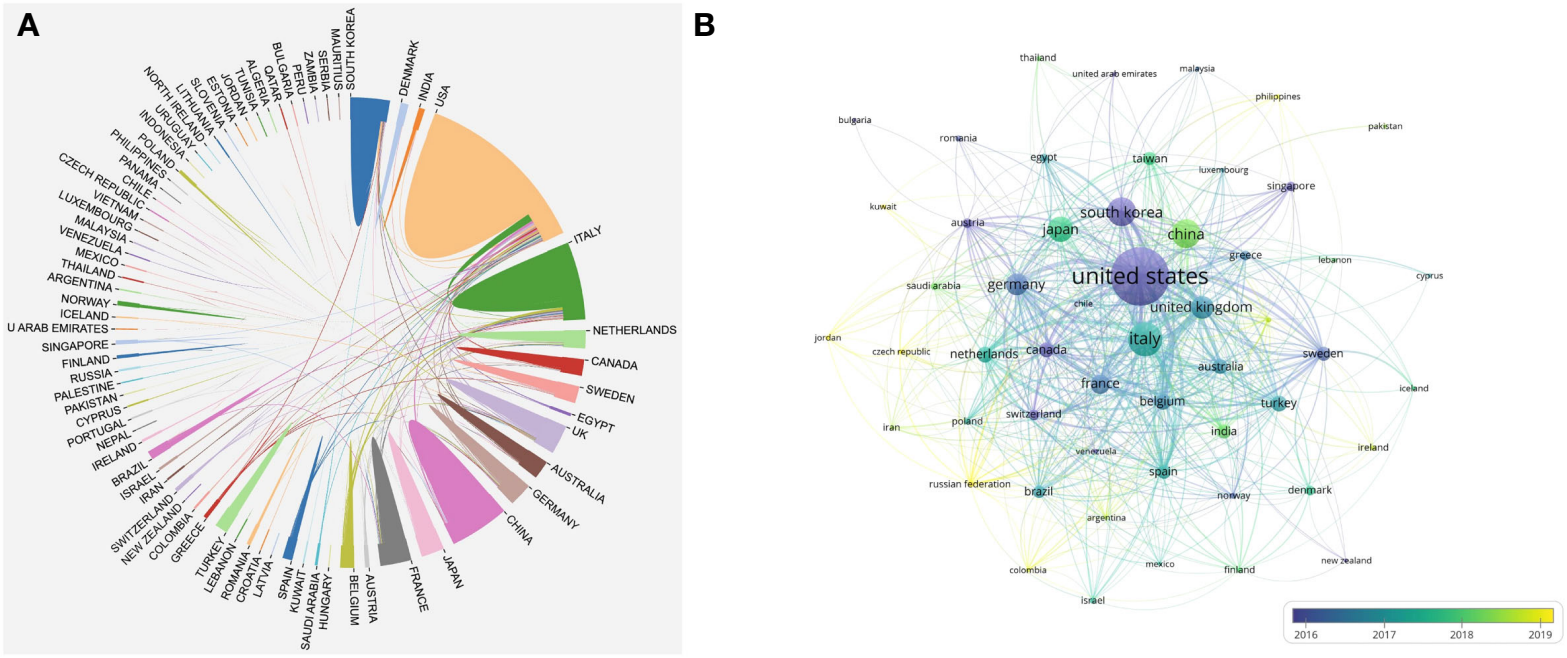
Knowledge map of national/regional collaborative network related to robotic surgery in oncology. **(A)** The map was produced by the Bibliometric Online Analysis Platform. **(B)** The size of the nodes is positively correlated with the number of countries/regions publications. The warmer the color of the nodes, the later the average publication time of the countries/regions. The lines between the nodes and the thickness of the lines represent the cooperation and cooperation intensity between the countries/regions. From: VOSviewer.

**Figure 3:**
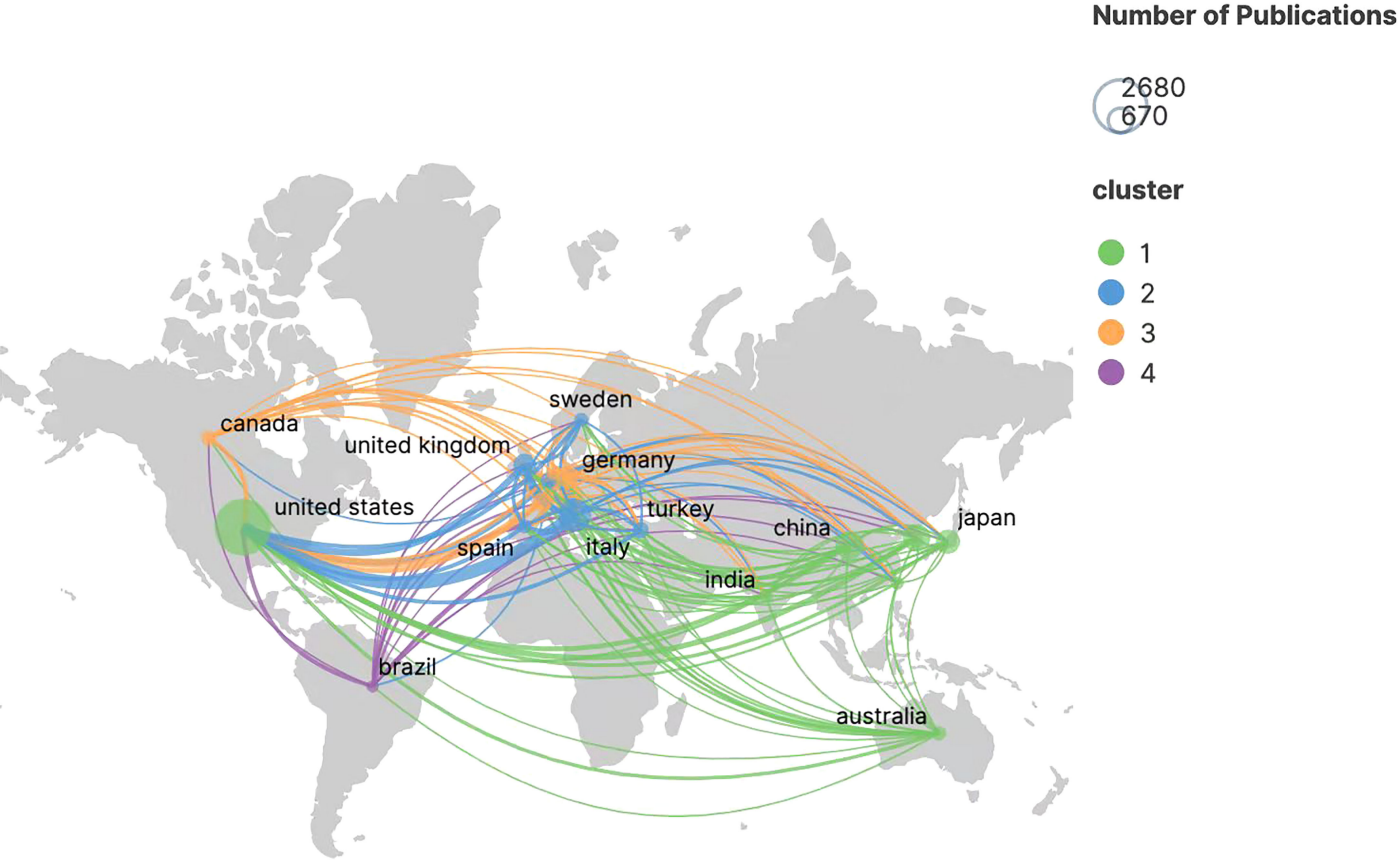
Geographical distribution of collaboration between countries/regions related to robotic surgery in oncology. The graph was generated by Scimago Graphica 1.0.24 software. The size of the circles is proportional to the number of publications. The color of the circles represents the different clusters, and the thickness of the lines represents the intensity of cooperation between countries/regions.

Over the past 20 years, a total of 81 countries/regions have published publications related to robotic surgery research in oncology. The United States was the major contributor to this research field with the most publications (2,678, accounting for 38.46% of all publications, 75,301 total citations, 28.12 average citations per paper, 118 H-index, and 1,267 total link strength), where it also ranked first in total citations, total link strength, and H-index. Each H-index refers to H publications published by journals, countries/regions, institutions, or authors, and each publication is cited at least H times. It is used to evaluate the academic influence of journals, countries/regions, institutions, or authors. The higher the H-index, the greater the academic influence (26–28). Followed by Italy (894, accounting for 12.84%, 23,426 total citations, 26.20 average citations per paper, 71 H-index, and 944 total link strength) and China (665, accounting for 9.55%, 9,701 total citations, 14.59 average citations per paper, 42 H-index, and 226 total link strength). These three countries contributed 60.85% of the number of publications and are the leading research countries in this field. Although Belgium ranked 10th in the number of publications (201, accounting for 2.89%, 7,543 total citations, 37.53 average citations per paper, 43 H-index, and 514 total link strength), the average citations per paper ranked first, indicating that the country’s publications are of high quality and are widely cited by researchers (**Table 1**). The map of the cooperation network among countries/regions is shown in **Figure 2**, and the geographical distribution map of countries/regions is shown in **Figure 3**. It can be seen from the figures that the United States and Italy cooperate more closely, and the cooperation between these two countries and other countries/regions is also more frequent. However, China is a highly productive country, but has less cooperation with other countries/regions and should strengthen exchanges and cooperation with other countries/regions.

**Table 1:** The top 10 countries/regions in the number of publications related to robotic surgery in oncology.

Rank	Countries/regions	Counts	Percentage	Total citations	Average citation per item	H-index	Total link strength
1	USA	2,678	38.46	75,301	28.12	118	1,267
2	Italy	894	12.84	23,426	26.20	71	944
3	China	665	9.55	9,701	14.59	42	226
4	South Korea	635	9.12	17,163	27.03	66	323
5	Japan	493	7.08	6,175	12.53	36	162
6	England	395	5.67	11,808	29.89	52	630
7	Germany	387	5.56	11,122	28.74	47	594
8	France	318	4.57	8,643	27.18	44	419
9	Turkey	216	3.10	2,959	13.70	29	221
10	Belgium	201	2.89	7,543	37.53	43	514

**Figure 4:**
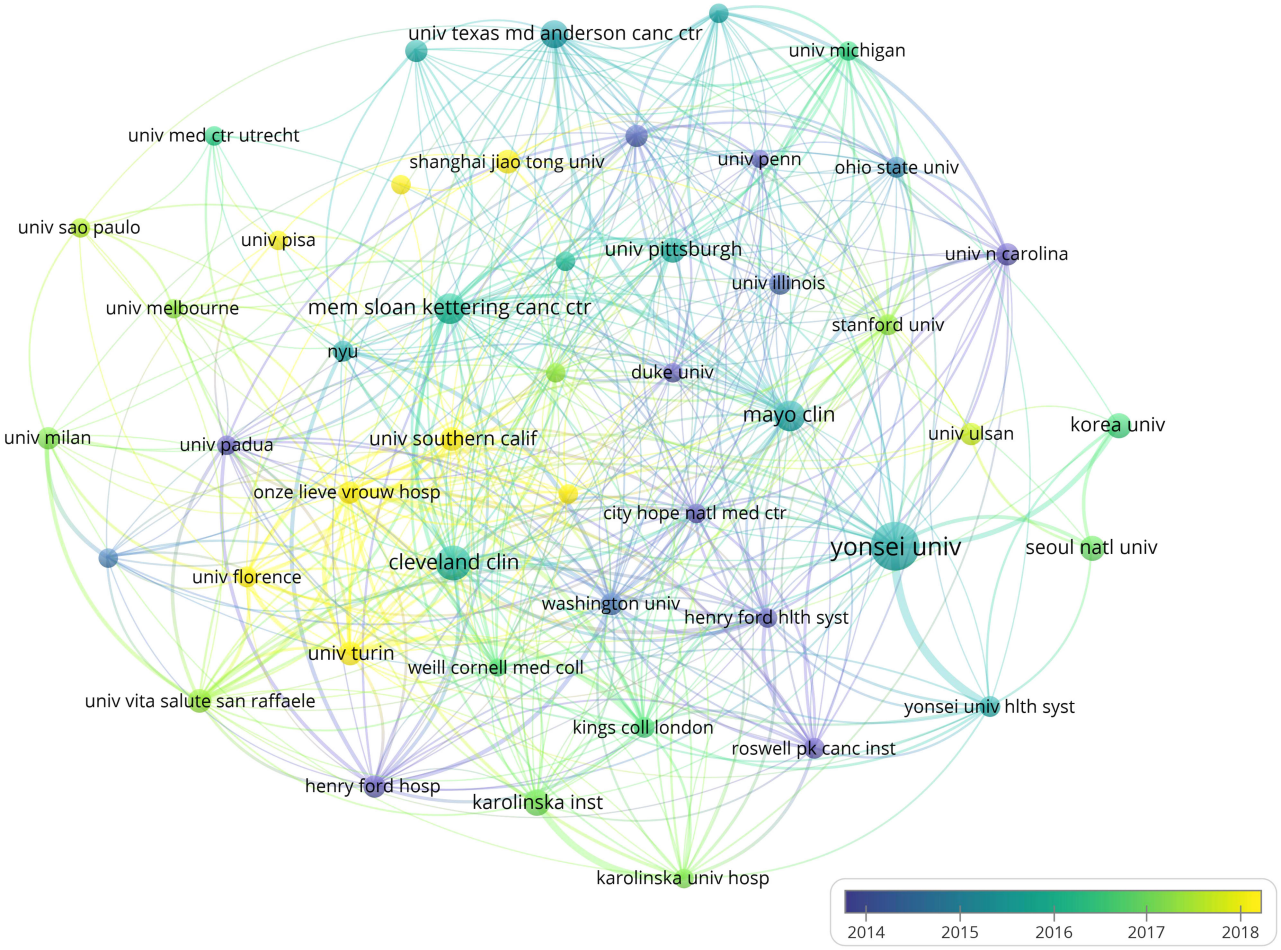
Knowledge map of the institutional collaboration network related to robotic surgery in oncology. The size of the nodes is proportional to the number of publications of the institution, and the warmer the color of the nodes, the later the average publication time of the institution. The lines between nodes represent the cooperation relationship between institutions, and the thickness of the lines is positively correlated with the intensity of cooperation. From: VOSviewer.

These 6,964 relevant studies were published by 4,071 institutions. The institutional collaboration network knowledge map shows institutions with no less than 40 publications (**Figure 4**). Among them, the top 5 institutions for publications were Yonsei University (256, 3.68%, 34.31 average citations per paper, 51 H-index, and 209 total link strength), Cleveland Clinic (134, 1.92%, 34.92 average citations per paper, 44 H-index, and 124 total link strength), Mayo Clinic (104, 1.49%, 47.35 average citations per paper, 38 H-index, and 138 total link strength), Memorial Sloan Kettering Cancer Center (103, 1.48%, 49.04 average citations per paper, 33 H-index, and 132 total link strength), and UTMD Anderson Cancer Center (86, 1.23%, 38.84 average citations per paper, 32 H-index, and 86 total link strength) (**Table 2**). Yonsei University in South Korea was the institution with the highest H-index and total link strength, while Memorial Sloan Kettering Cancer Center in the United States ranked first in average citations per paper. It shows that Yonsei University has a high academic influence in this field and cooperates closely with other research institutions, while the papers published by Memorial Sloan Kettering Cancer Center are of high research quality and widely cited. 50% of the top 10 institutions in publications were from the United States and 30% were from South Korea, indicating that institutions in the United States and South Korea have made outstanding contributions in this research field. However, China, as a highly productive country, has no top-ranked research institutions, indicating that Chinese research institutions lack core competitiveness in the world and need to further strengthen their own research strength and close cooperation with other international research institutions in order to enhance the influence of Chinese research institutions in the international arena.

**Table 2:** The top 10 institutions for the number of publications related to oncology robotic surgery.

Rank	Institutions	Counts	Percentage	Average citation per item	H-index	Countries/regions	Total link strength
1	Yonsei University	256	3.68	34.31	51	South Korea	209
2	Cleveland Clinic	134	1.92	34.92	44	USA	124
3	Mayo Clinic	104	1.49	47.35	38	USA	138
4	Memorial Sloan Kettering Cancer Center	103	1.48	49.04	33	USA	132
5	UTMD Anderson Cancer Center	86	1.23	38.84	32	USA	86
6	Karolinska Institutet	83	1.19	36.15	37	Sweden	120
7	korea university	71	1.02	23.58	25	South Korea	62
8	Seoul National University	68	0.98	27.70	23	South Korea	60
9	University of Southern California	67	0.96	32.03	31	USA	114
10	Vita Salute San Raffaele University	62	0.89	44.15	30	Italy	165

### Analysis of journals and cited journals

A total of 850 journals published papers related to robotic surgery in oncology. Among them, the *Journal of Robotic Surgery* published the most publications (n = 323(4.64%), average citations per item = 6.65, H-index = 18, IF2021 = 2.484, Q2), followed by *Surgical Endoscopy and Other Interventional Techniques* (n = 267(3.83%), average citations per item = 33.92, H-index = 53, IF2021 = 3.453, Q2), *BJU Interventional* (n = 220(3.16%), average citations per item = 35.07, H-index = 47, IF2021 = 5.969, Q1), *Journal of Endourology* (n = 220(3.16%), average citations per item = 16.05, H-index = 32, IF2021 = 2.619, Q3), and *European Urology* (n = 200(2.87%), average citations per item = 89.55, H-index = 81, IF2021 = 24.267, Q1) (**Table 3**). The average impact factor (IF) and H-index of the top 10 productivity journals were 5.798 and 37.9, respectively. *European Urology* ranked first in average citation per item, H-index, and IF, which indicates that it is a high-quality core journal and also a highly cited journal in this field. Of the top 10 journals in terms of publications, 30% belonged to Q1 and 40% to Q2, indicating a high level of research in the field. Among these journals, 30% were from the United Kingdom and 50% were from the United States, which shows that American and British journals have made outstanding contributions in this field and are deeply concerned by researchers.

**Table 3:** The top 10 productive journals related to robotic surgery in oncology.

Rank	Journal	Counts	Percentage	Average citation per item	H-index	IF(2021)	Quartile in category
1	J ROBOT SURG (England)	323	4.64	6.65	18	2.484	Q2
2	SURG ENDOSC (United States)	267	3.83	33.92	53	3.453	Q2
3	BJU INT (England)	220	3.16	35.07	47	5.969	Q1
4	J ENDOUROL (United States)	220	3.16	16.05	32	2.619	Q3
5	EUR UROL (Netherlands)	200	2.87	89.55	81	24.267	Q1
6	INT J MED ROBOT COMP (England)	171	2.46	14.27	26	2.483	Q3
7	UROLOGY (United States)	131	1.88	37.95	38	2.633	Q3
8	J UROLOGY (United States)	129	1.85	51.13	45	7.600	Q1
9	WORLD J UROL (United States)	114	1.64	16.41	25	3.661	Q2
10	ASIAN J SURG (China)	84	1.21	7.20	14	2.808	Q2

**Figure 5:**
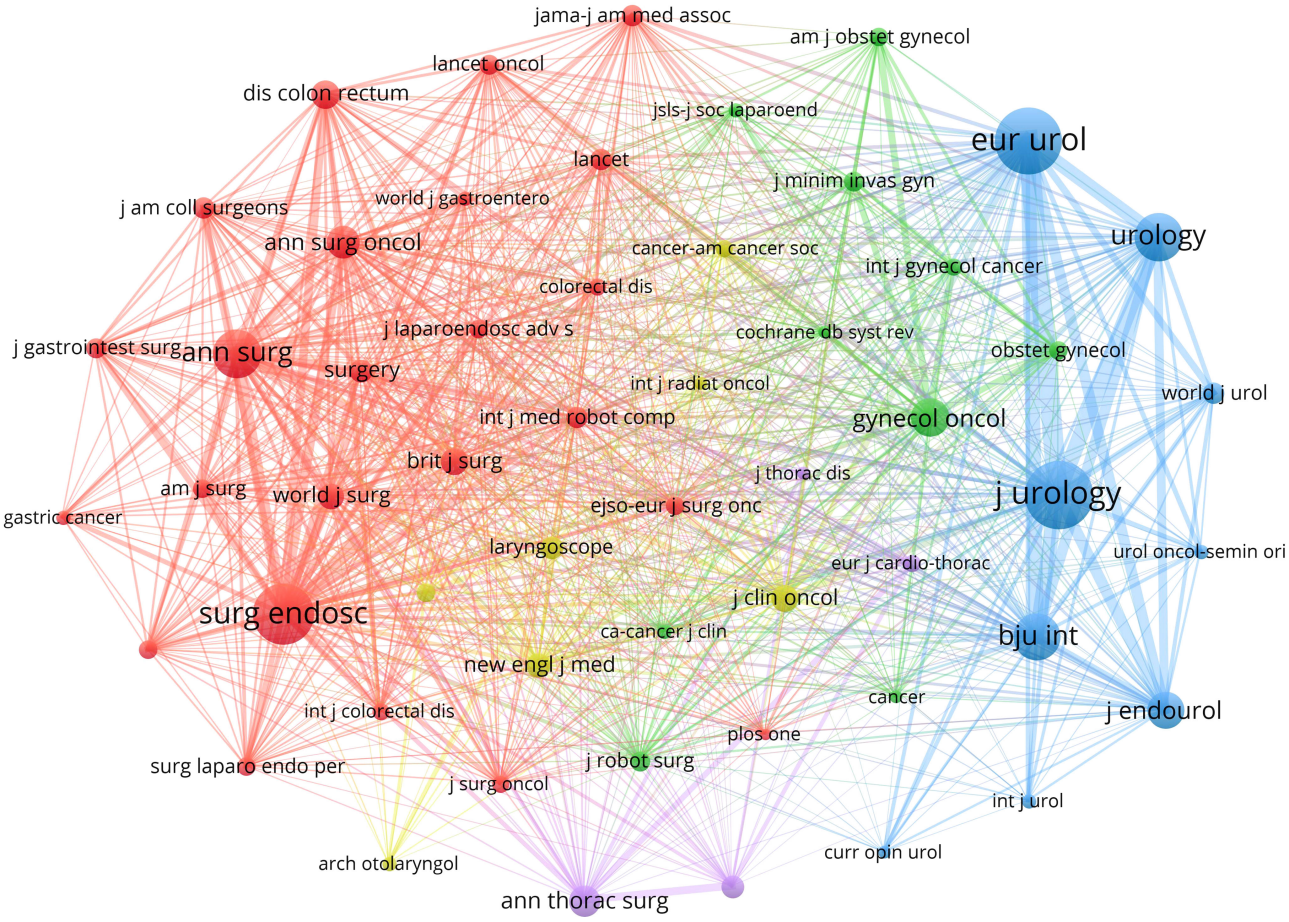
Knowledge map of co-cited journals network related to robotic surgery in oncology. The size of the nodes is positively related to the citation frequency of the journal. The color of the nodes represents different clusters. The lines between the nodes represent the co-citation relationship between journals. The thickness of the lines is directly proportional to the co-citation strength. From: VOSviewer.

Journals with at least 600 citations as indicated by the journal co-citation network knowledge map (**Figure 5**). Journal co-citation refers to the phenomenon that two or more journals are cited by the same document, which reveals the relevance between various journals and disciplines, and can obtain the distribution of knowledge base in this field (29). The most frequently cited journal was *Journal of Urology* (n = 13,712, total link strength = 359,201, H-index = 45, IF2021 = 7.600, Q1), followed by *European Urology* (n = 13,333, total link strength = 347,684, H-index = 81, IF2021 = 24.267, Q1), *Surgical Endoscopy and Other Interventional Techniques* (n = 11,176, total link strength = 340,950, H-index = 53, IF2021 = 3.453, Q2), *Urology* (n = 7,276, total link strength = 218,578, H-index = 38, IF2021 = 2.633, Q3), and *Annals of Surgery* (n = 7,176, total link strength = 226,129, H-index = 28, IF2021 = 13.787, Q1) (**Table 4**). Among them, the *Journal of Urology* has close co-citation relationships with *European Urology, Urology, BJU Interventional, Journal of Endourology, Annals of Surgery*, and *Surgical Endoscopy and Other Interventional Techniques*, etc.; *Surgical Endoscopy and Other Interventional Techniques* has strong co-citation relationships with *Annals of Surgery, Annals of Surgical Oncology, Annals of Thoracic Surgery, Gynecologic Oncology, European Urology, Urology, Journal of Urology, BJU Interventional*, and *Journal of Endourology*, etc. 80% of the top 10 co-cited journals were from the United States, again indicating that American journals have important academic prestige in this field and are widely recognized by many scholars. *European Urology* was the journal with the highest H-index and IF, which again indicates that it is a high-quality core journal in this research field. In addition, the average H-index and IF of the 10 co-cited journals were 42.4 and 7.507, respectively, with 70% being Q1, indicating that high-quality journals are cited more frequently. Therefore, we should pay more attention to the highly cited journals when reading the literature.

**Table 4:** The top 10 co-cited journals in citation frequency related to robotic surgery in oncology.

Rank	Co-cited Journal	Citation frequency	Total link strength	H-index	IF(2021)	Quartile in category
1	J UROLOGY (United States)	13,712	359,201	45	7.600	Q1
2	EUR UROL (Netherlands)	13,333	347,684	81	24.267	Q1
3	SURG ENDOSC (United States)	11,176	340,950	53	3.453	Q2
4	UROLOGY (United States)	7,276	218,578	38	2.633	Q3
5	ANN SURG (United States)	7,176	226,129	28	13.787	Q1
6	BJU INT (England)	7,101	218,058	47	5.969	Q1
7	GYNECOL ONCOL (United States)	4,800	97,785	40	5.304	Q1
8	J ENDOUROL (United States)	4,396	139,034	32	2.619	Q3
9	ANN SURG ONCOL (United States)	3,736	125,756	32	4.339	Q1
10	ANN THORAC SURG (United States)	3,260	69,756	28	5.102	Q1

**Figure 6:**
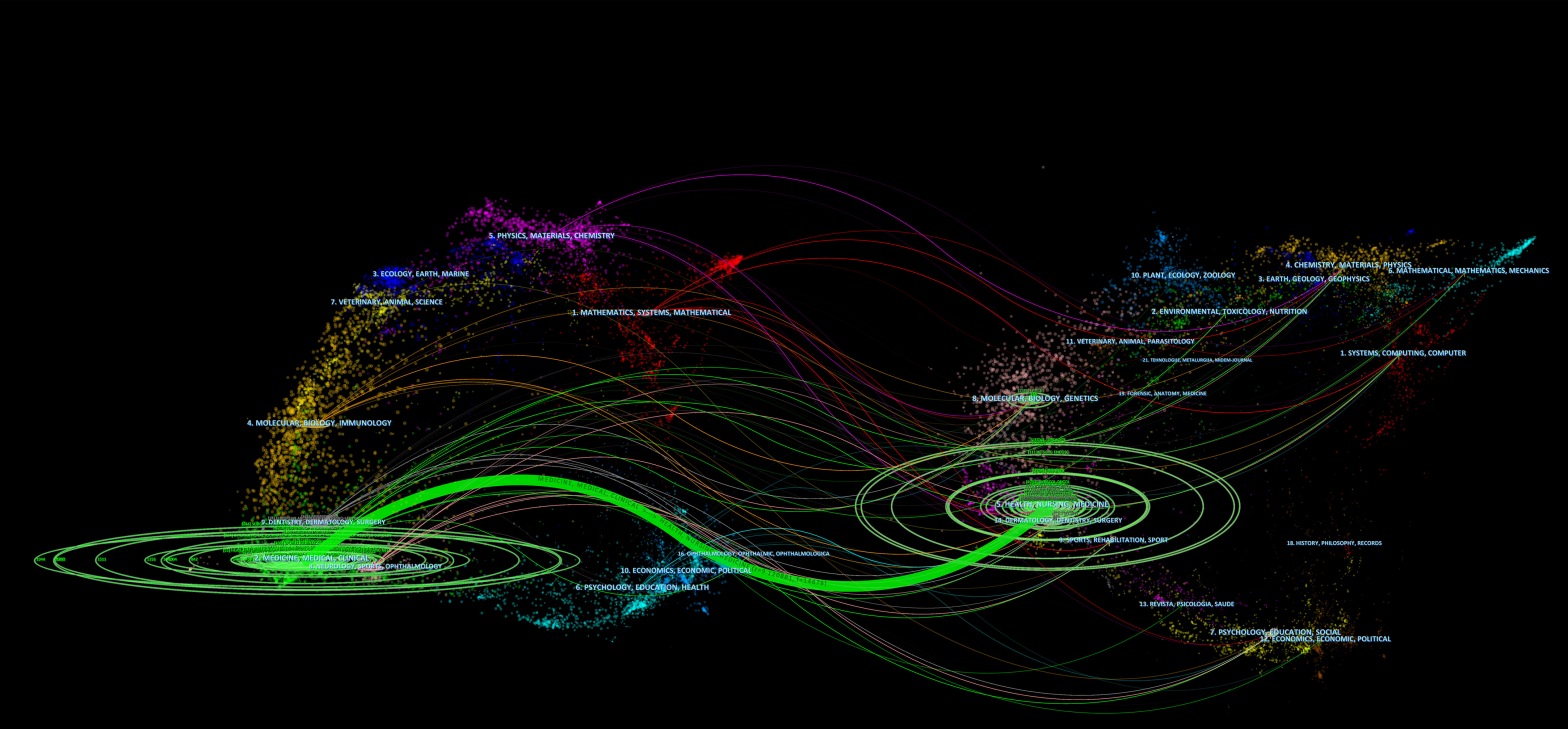
The dual-map overlay of journals related to robotic surgery in oncology. The citing journals are on the left of the map, and the cited journals are on the right, and the colored paths represent citation relationships.

The dual-map overlay of journals can visually display the distribution of journals and the citation relationship between citing journals and cited journals. The left side of the map is the citing journals, the right side is the cited journals, and the colored paths represent the citation relationship (30, 31). A green main citation path is identified in **Figure 6**, which indicates that papers published in Health/Nursing/Medicine journals were mainly cited by papers published in Medicine/Medical/Clinical journals.

### Analysis of authors and cited authors

**Figure 7:**
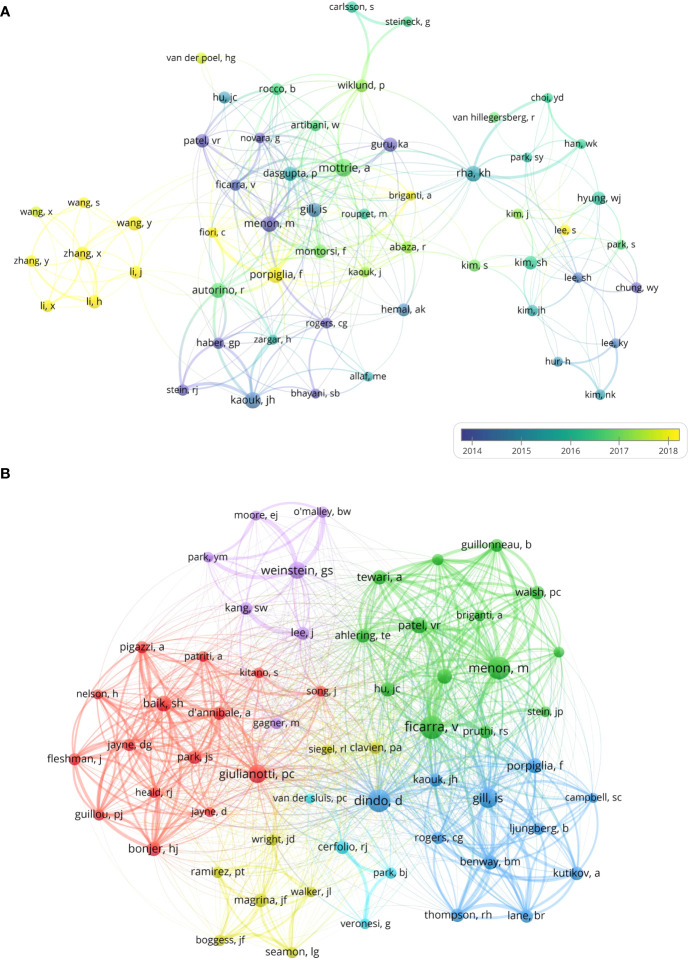
Knowledge map of authors cooperation and co-cited authors network related to robotic surgery in oncology. **(A)** knowledge map of authors cooperation network. The size of the nodes represents the number of papers published by the author. The warmer the color of the nodes, the later the author’s average publishing time. The lines between the nodes represent the cooperation between authors, and the thickness of the lines represents the strength of cooperation. **(B)** knowledge map of co-cited authors. The size of the nodes is proportional to the citation frequency of the author. The color of the nodes represents different clusters, and the lines between the nodes and the thickness of the lines represent the co-citation relationship and co-citation strength between authors. From: VOSviewer.

A total of 22,846 authors conducted related research in the field of oncology robotic surgery. The knowledge map of the authors cooperation network shows the authors with no less than 25 publications (**Figure 7A**). The top 5 authors in the publication were Mottrie A (97, 1.39%, 43.25 average citation per publication, 32 H-index, and 229 total link strength), Rha KH (75, 1.08%, 26.75 average citation per publication, 23 H-index, and 92 total link strength), Porpiglia F (73, 1.05%, 27.18 average citation per publication, 27 H-index, and 219 total link strength), Kaouk JH (72, 1.03%, 37.35 average citation per publication, 27 H-index, and 126 total link strength), and Menon M (72, 1.03%, 85.24 average citation per publication, 39 H-index, and 97 total link strength) (**Table 5**). Among them, Mottrie A, from the Department of Urology, Onze Lieve Vrouw Hospital, Aalst, Belgium, was the author with the most publications and has made important contributions in this field. The results of a recent study by him demonstrated that the robot-assisted radical prostatectomy (RARP) metric, which is critical for efficient and quality-assured surgical training, reliably distinguished between very experienced surgeons (VES) and novices for objectively scoring procedural performance (32). Menon M from Henry Ford Hospital in the United States had the highest average citation per publication and H-index, indicating that his research level is very high and his academic achievements are widely recognized and cited by scholars. His research team developed a nerve-sparing robot-assisted radical cystoprostatectomy (RRCP) technique using the da Vinci system that allowed precise and rapid removal of the bladder with minimal blood loss (33). Mottrie A and Menon M have active partnerships with Rha KH, Porpiglia F, Ficarra V, Montorsi F, Dasgupta P, Guru KA, Gill IS, Patel VR, Autorino R, Novara G, Kaouk JH, etc. In addition, 90% of the top 10 authors in terms of the number of publications were from Europe or the United States, especially 50% from the United States, which indicates that the main research contributions come from Europe and the United States. These researchers have made important contributions and have high academic influence in this field.

**Table 5:** The top 10 authors in number of publications related to robotic surgery in oncology.

Rank	Author	Counts	Percentage	Average citation per item	H-index	Countries/regions	Total link strength
1	Mottrie A	97	1.39	43.25	32	Belgium	229
2	Rha KH	75	1.08	26.75	23	South Korea	92
3	Porpiglia F	73	1.05	27.18	27	Italy	219
4	Kaouk JH	72	1.03	37.35	27	USA	126
5	Menon M	72	1.03	85.24	39	USA	97
6	Autorino R	70	1.01	27.70	27	USA	167
7	Dasgupta P	62	0.89	38.89	27	England	82
8	Gill IS	60	0.86	47.00	29	USA	70
9	Guru KA	54	0.78	35.76	23	USA	35
10	Montorsi F	54	0.78	56.31	23	Italy	141

The authors’ co-cited network knowledge map shows authors whose citation frequency is not less than 200 times (**Figure 7B**). Author co-citation refers to the phenomenon of two or more authors being co-cited in the same piece of literature, which can reveal the academic community in the field and identify highly influential research groups (34, 35). The top 5 co-cited authors in terms of citation frequency were Ficarra V (952 times, 5,102 total link strength, and 24 H-index), Menon M (891 times, 5,668 total link strength, and 39 H-index), Dindo D (871 times, 2,945 total link strength, and 21 H-index), Gill IS (689 times, 4,247 total link strength, and 29 H-index), and Giulianotti PC (593 times, 1,988 total link strength, and 13 H-index) (**Table 6**). Among them, Ficarra V, from the University of Messina, Italy, was the most frequently cited author, whose a recent study demonstrated the use of a novel urethral fixation technique compared with standard vesicourethral anastomosis in robot-assisted radical prostatectomy (RARP), early urinary continence recovery was significantly improved, and there was no significant difference in operating room time or perioperative complications (36). Menon M was not only a highly productive author, but also a highly cited author, with the highest average citation per publication, H-index, and total link strength, again demonstrating his high academic influence and outstanding contribution in this research field. It is noteworthy that 60% of the top 10 cited authors were from the United States and 30% were from Italy, indicating that authors from the United States and Italy have high academic influence in this field, and their research results are recognized and widely cited by researchers. On the contrary, although China is a highly productive country, the individual academic influence of researchers is insufficient, and it is necessary to strengthen international exchanges and cooperation to enhance individual scientific research strength.

**Table 6:** The top 10 co-cited authors in terms of citation frequency related to robotic surgery in oncology.

Rank	Co-cited Author	Citation frequency	Total link strength	H-index	Countries/regions
1	Ficarra V	952	5,102	24	Italy
2	Menon M	891	5,668	39	USA
3	Dindo D	871	2,945	21	Switzerland
4	Gill IS	689	4,247	29	USA
5	Giulianotti PC	593	1,988	13	USA
6	Weinstein GS	533	1,986	14	USA
7	Patel VR	474	2,992	20	USA
8	Novara G	421	2,060	23	Italy
9	Porpiglia F	401	2,075	27	Italy
10	Tewari A	388	2,797	21	USA

### Analysis of cited references

**Figure 8:**
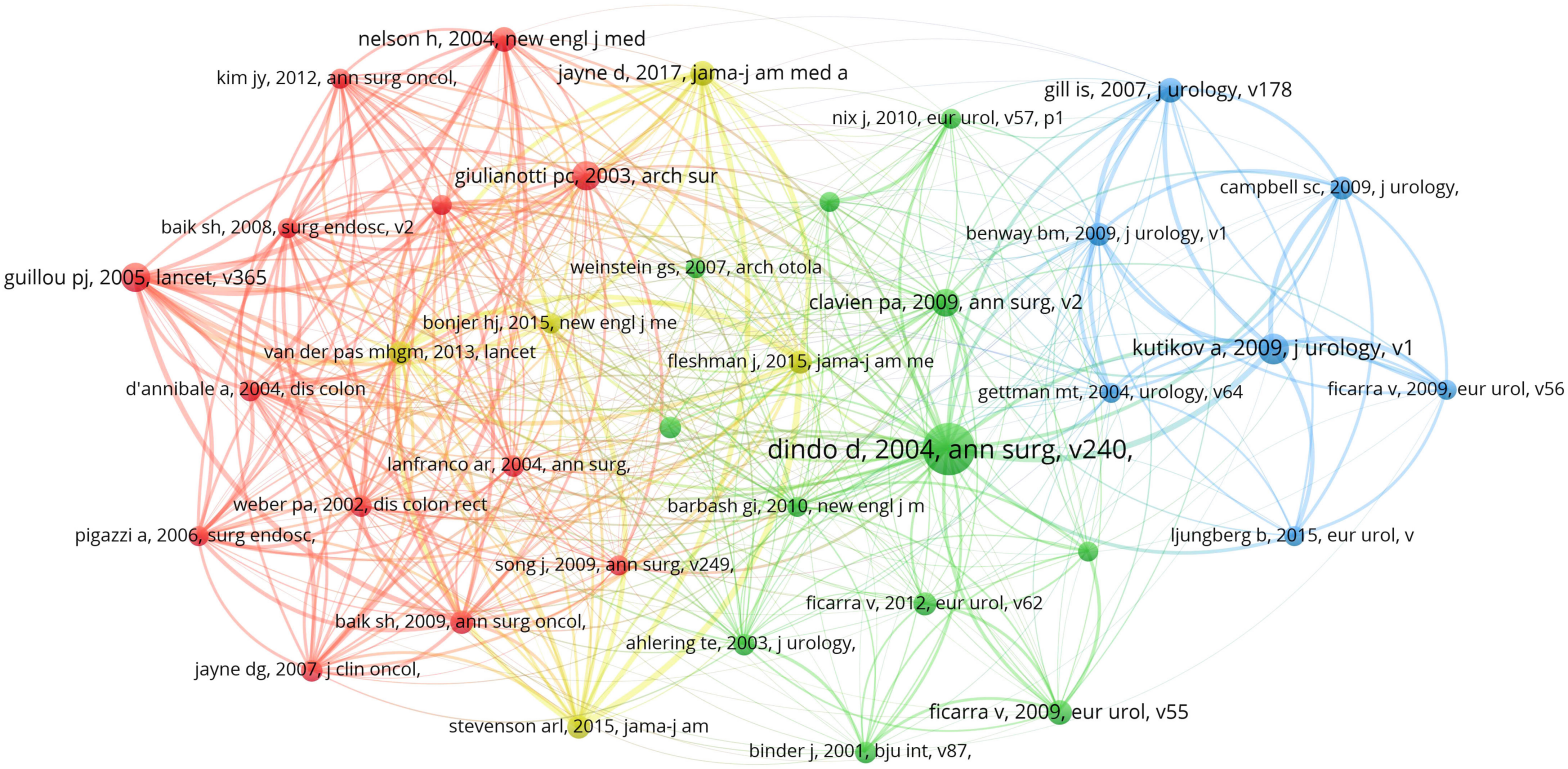
knowledge map of co-cited references network related to robotic surgery in oncology. The size and color of the nodes represent the citation frequency and different clustering, and the lines of the nodes and the thickness of the lines represent the co-citation relationship and co-citation strength of the references. From: VOSviewer.

The references co-citation network knowledge map shows co-cited literature with at least 120 citations (**Figure 8**). Document co-citation refers to the phenomenon that two or more papers are cited by the same study. By analyzing the key nodes in the co-citation network, we can reveal the knowledge structure of this field, explore the knowledge base and research frontier in this field, and find documents with high academic influence and play a key role (37, 38). The top 10 most frequently cited papers are listed in **Table 7**. The average IF and H-index of the journals that published these 10 papers (excluding one paper with no relevant information) were 67.503 and 404.11, respectively, and these papers were all Q1, indicating that these are high-quality papers and are the basis of research in the field. The most frequently cited paper was published in *Annals of Surgery* by Dindo D et al. (39) in 2004. The study reevaluated and modified the postoperative complications grading system and proved its reliability. The *Journal of Urology* published the second co-cited publication by Kutikov A et al. (40) in 2009. The authors’ team developed a standardized nephrometry scoring system to quantify the salient anatomy of renal masses. In 2005, Guillou PJ et al. (41) published the third co-cited study in the *Lancet.* This study confirmed that laparoscopic-assisted colorectal cancer surgery can achieve the same short-term efficacy compared with open surgery through a multicenter randomized controlled trial. The fourth co-cited paper was published by Giulianotti PC et al. (42) in the *Archives of Surgery* in 2003. This study demonstrated the safety and feasibility of robotic technology as minimally invasive surgery in clinical applications. In 2009, the fifth co-cited study was published by Clavien PA et al. (43) in the *Annals of Surgery.* This study demonstrated the validity and applicability of the classification in many surgical fields by assessing the Clavien-Dindo classification of surgical complications. *JAMA-Journal of the American Medical Association* published the sixth co-cited publication by Jayne D et al. (44) in 2017. This study found that robotic-assisted laparoscopic surgery did not significantly reduce the probability of conversion to open surgery by comparing robotic-assisted and conventional laparoscopic rectal adenocarcinoma resection. The seventh co-cited paper was published by Nelson H et al. (45) in the *New England Journal of Medicine* in 2004. A comparative study of laparoscopic-assisted colectomy and open colectomy showed that there was no significant difference in postoperative tumor recurrence rate between the two groups. *European Urology* published the eighth co-cited publication by Ficarra V et al. (46) in 2009. This study systematically reviewed the comparative studies of retropubic, laparoscopic, and robot-assisted radical prostatectomy in terms of perioperative, functional, and oncologic outcomes. The ninth co-cited paper was published by Gill IS et al. (47) in the *Journal of Urology* in 2007. By comparing laparoscopic partial nephrectomy with open partial nephrectomy, this study found that laparoscopic partial nephrectomy had the advantages of shorter operation time, less intraoperative bleeding, and shorter hospital stay. In 2009, Baik SH et al. (48) published the tenth co-cited study in the *Annals of Surgical Oncology*. This study, by comparing robotic-assisted low anterior resection (R-LAR) using the Da Vinci surgical system with standard laparoscopic low anterior resection (L-LAR), found that R-LAR using the Da Vinci surgical system was safe, effective, and achieved satisfactory perioperative outcomes. Through the analysis of these 10 papers, the classification of surgical complications, laparoscopic surgery, open surgery, colorectal cancer surgery, robotic-assisted surgery, prostatectomy, partial nephrectomy, perioperative outcomes, and functional and oncologic outcomes are the basis of oncologic robotic surgery research.

**Table 7:** The top 10 co-cited references in terms of citation frequency related to robotic surgery in oncology.

Rank	Co-cited reference	Author and publication year	Citations	Total link strength	Journal IF(2021)	H-index	Quartile in category
1	Classification of surgical complications: a new proposal with evaluation in a cohort of 6336 patients and results of a survey	Dindo D, 2004	848	1,423	ANN SURG (IF:13.787)	284	Q1
2	The R.E.N.A.L. nephrometry score: a comprehensive standardized system for quantitating renal tumor size, location and depth	Kutikov A, 2009	285	679	J UROLOGY (IF:7.600)	236	Q1
3	Short-term endpoints of conventional versus laparoscopic-assisted surgery in patients with colorectal cancer (MRC CLASICC trial): multicentre, randomised controlled trial	Guillou PJ, 2005	264	1,397	LANCET (IF:202.731)	700	Q1
4	Robotics in general surgery: personal experience in a large community hospital	Giulianotti PC, 2003	254	555	ARCH SURG-CHICAGO (IF : NA)	NA	NA
5	The Clavien-Dindo classification of surgical complications: five-year experience	Clavien PA, 2009	245	422	ANN SURG (IF:13.787)	284	Q1
6	Effect of Robotic-Assisted *vs* Conventional Laparoscopic Surgery on Risk of Conversion to Open Laparotomy Among Patients Undergoing Resection for Rectal Cancer: The ROLARR Randomized Clinical Trial	Jayne D, 2017	199	816	JAMA-J AM MED ASSOC (IF:157.335)	622	Q1
7	A comparison of laparoscopically assisted and open colectomy for colon cancer	Nelson H, 2004	191	888	NEW ENGL J MED (IF:176.079)	933	Q1
8	Retropubic, laparoscopic, and robot-assisted radical prostatectomy: a systematic review and cumulative analysis of comparative studies	Ficarra V, 2009	187	367	EUR UROL (IF:24.267)	187	Q1
9	Comparison of 1,800 laparoscopic and open partial nephrectomies for single renal tumors	Gill IS, 2007	182	503	J UROLOGY (IF:7.600)	236	Q1
10	Robotic versus laparoscopic low anterior resection of rectal cancer: short-term outcome of a prospective comparative study	Baik SH, 2009	171	912	ANN SURG ONCOL (IF:4.339)	155	Q1

NA, not available.

### References with citation bursts

**Figure 9:**
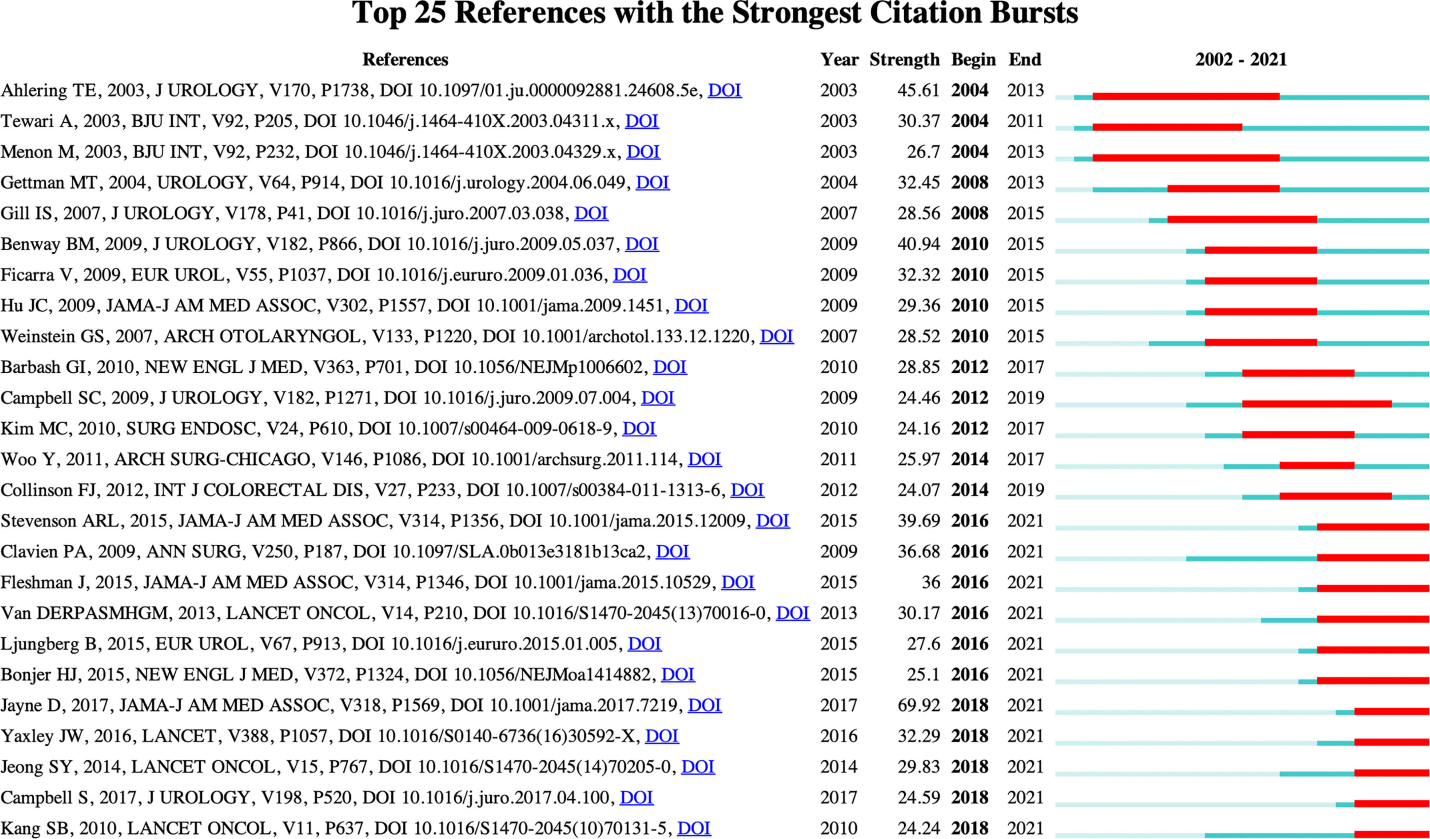
The top 25 references with the strongest citation bursts. The blue line indicates the time interval, and the red line indicates the time from the begin to the end of the citation burst.

A citation burst refers to papers whose citation frequency increases suddenly in a short time. By analyzing the citation burst, the research hotspots and emerging trends in this field can be found (49, 50). The top 25 references with the strongest citation bursts were detected by setting the minimum duration of the burst to 3 years (**Figure 9**). The blue bar represents the timeline, and the red bar indicates the time from the start to the end of the citation burst. The “strength” represents the burst strength. The higher the value, the greater the burst strength (51). Among these 25 references, the citation burst of 11 references ended in 2021. They reflect the latest research trends and are therefore further analyzed. The first reference with the greatest burst strength was published by Jayne D et al. (44) in *JAMA-Journal of the American Medical Association* in 2017. This study found that robotic-assisted laparoscopic surgery did not significantly reduce the probability of conversion to open surgery by comparing robotic-assisted and conventional laparoscopic rectal adenocarcinoma resection. In 2015, the paper with the second highest burst strength of the 11 publications with the citation burst was published in *JAMA-Journal of the American Medical Association* by Stevenson ARL et al. (52). Through the comparative analysis of laparoscopic rectal resection and open rectal resection in the treatment of T1-T3 rectal adenocarcinoma patients, this study found that the noninferiority of laparoscopic surgery in successful resection was not established. Clavien PA et al. (43) published the study with the third highest citation burst in *Annals of Surgery* in 2009. This study demonstrated the validity and applicability of the classification in many surgical fields by assessing the Clavien-Dindo classification of surgical complications. The publication with the fourth highest citation burst was published by Fleshman J et al. (53) in *JAMA-Journal of the American Medical Association* in 2015. In this study, 486 patients with clinical stage II or III rectal cancer were randomized to either laparoscopic or open resection. The authors found that laparoscopic rectal cancer resection did not meet the criteria for noninferiority of pathological outcomes compared with open resection. The *Lancet* published the paper with the fifth highest citation burst by Yaxley JW et al. (54) in 2016. This study analyzed robotic-assisted laparoscopic prostatectomy and radical retropubic prostatectomy through a clinical randomized controlled trial and found that the two techniques achieved similar functional outcomes at 12 weeks postoperatively. In 2013, the study with the sixth highest citation burst was published in *Lancet Oncology* by van der Pas MH et al. (55). This study confirmed through a multicenter clinical randomized controlled trial that laparoscopic rectal cancer resection had similar outcomes in terms of safety, resection margins, and completeness of resection compared to open resection. Jeong SY et al. (56) published the study with the seventh highest citation burst in *Lancet Oncology* in 2014. This study demonstrated that laparoscopic resection results in similar disease-free survival outcomes compared with open resection in the treatment of patients with mid-rectal or low-rectal cancer who received preoperative chemoradiotherapy. The publication with the eighth highest citation burst was published by Ljungberg B et al. (57) in *European Urology* in 2015. This review presented the Renal Cell Carcinoma (RCC) guidelines updated in 2014, providing the best and most reliable contemporary evidence base for RCC management. The *New England Journal of Medicine* published the paper with the ninth highest citation burst by Bonjer HJ et al. (58) in 2015. This trial, comparing laparoscopic surgery and open surgery for solitary adenocarcinoma of the rectum, demonstrated similar outcomes for locoregional recurrence, disease-free survival, and overall survival. The publication with the tenth highest citation burst was published by Campbell S et al. (59) in the *Journal of Urology* in 2017. This paper systematically reviewed the AUA guidelines and pointed out that factors such as treatment effectiveness and potential morbidities, oncological issues, and functional outcomes should be considered in the counseling/management of patients with clinically localized renal masses. Finally, in 2010, Kang SB et al. (60) published the study with the eleventh highest citation burst in *Lancet Oncology*. This study found that laparoscopic surgery was safe and had short-term benefits and the same quality of oncological resection compared with open surgery in the treatment of patients with mid and low rectal cancer after chemoradiotherapy. According to the analysis of the literature above, colorectal neoplasms and urologic neoplasms are the main fields of robotic surgery research. Because of the particularity of oncological diseases and the insignificant therapeutic advantage of robotic surgery in oncology, the research trend of robotic surgery for tumors is to further evaluate the pathological outcomes, functional and oncological outcomes, survival rate, quality of life, and other indicators of robotic surgery in the medium and long term through clinical randomized controlled trials with laparoscopic and open surgery.

### Analysis of keywords

**Figure 10:**
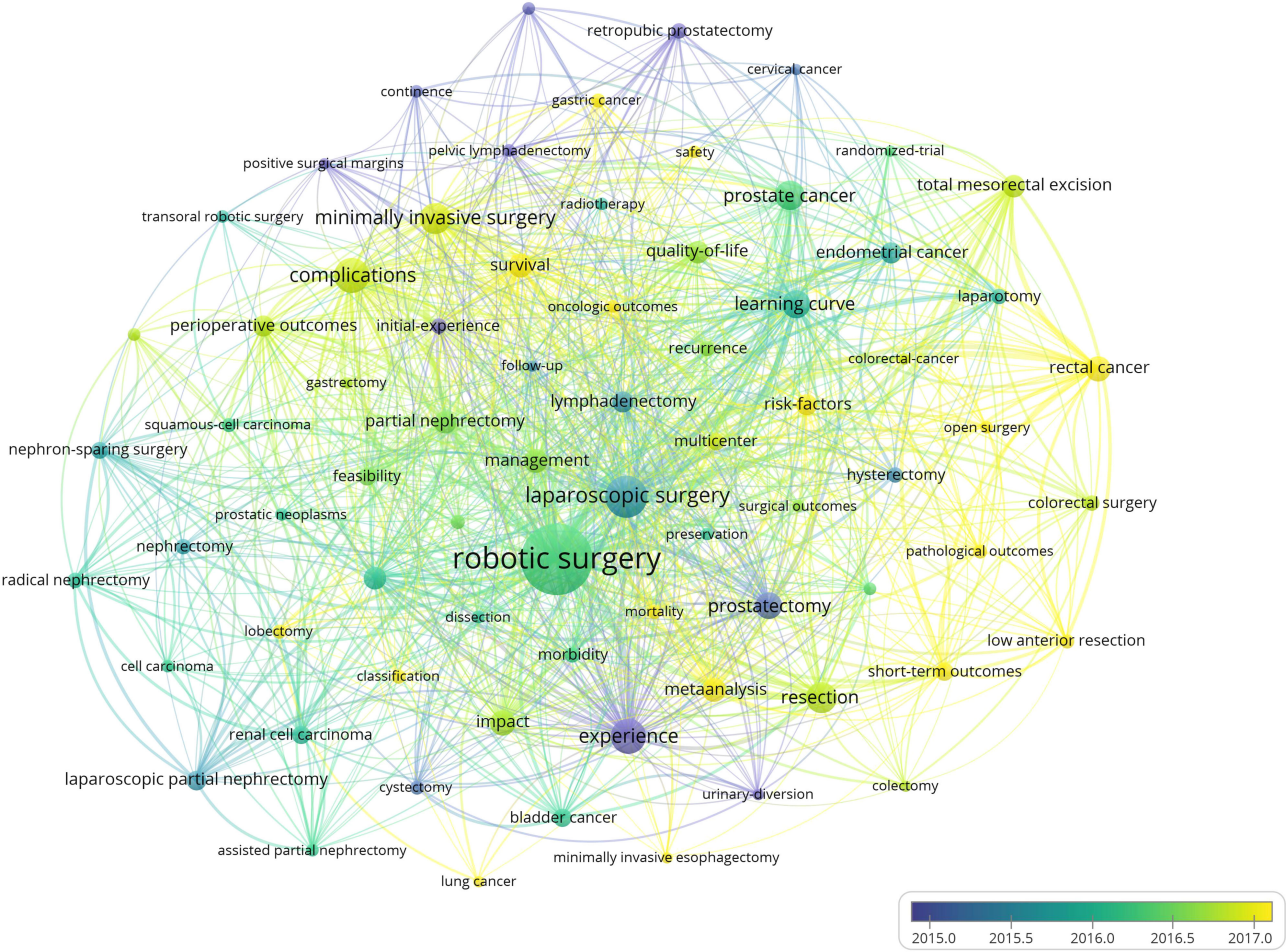
knowledge map of keywords co-occurrence network related to robotic surgery in oncology. The size of the nodes is proportional to the frequency of keywords occurrence. The warmer the color of the nodes, the later the average keywords appearance time. The lines between the nodes and the thickness of the lines represent the co-occurrence and co-occurrence strength of the keywords. From: VOSviewer.

**Figure 11:**
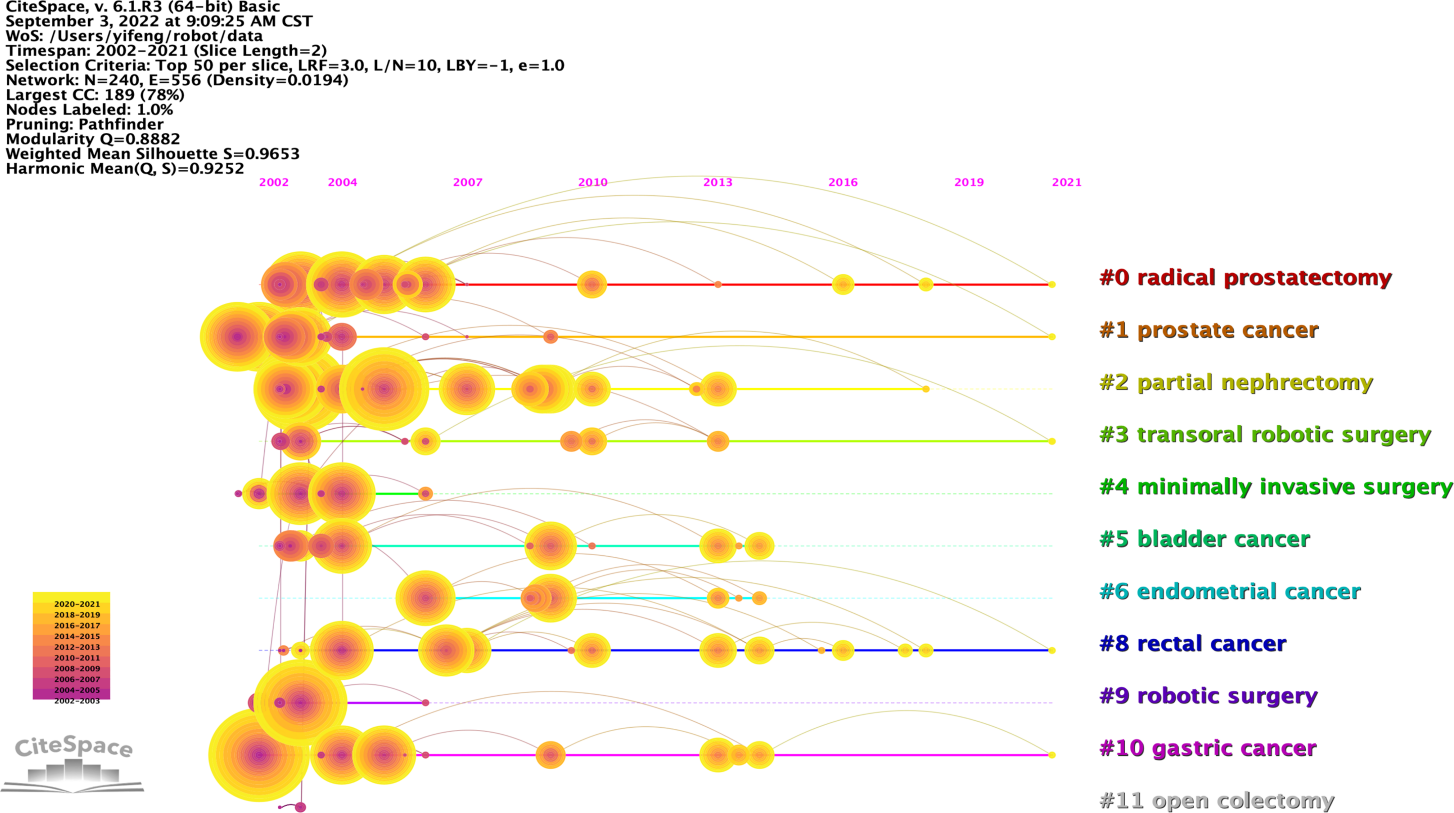
Timeline knowledge map of keywords related to robotic surgery in oncology. The keywords of the same cluster are placed on the same horizontal line, and the more to the right, the later the keyword appears.

**Figure 12:**
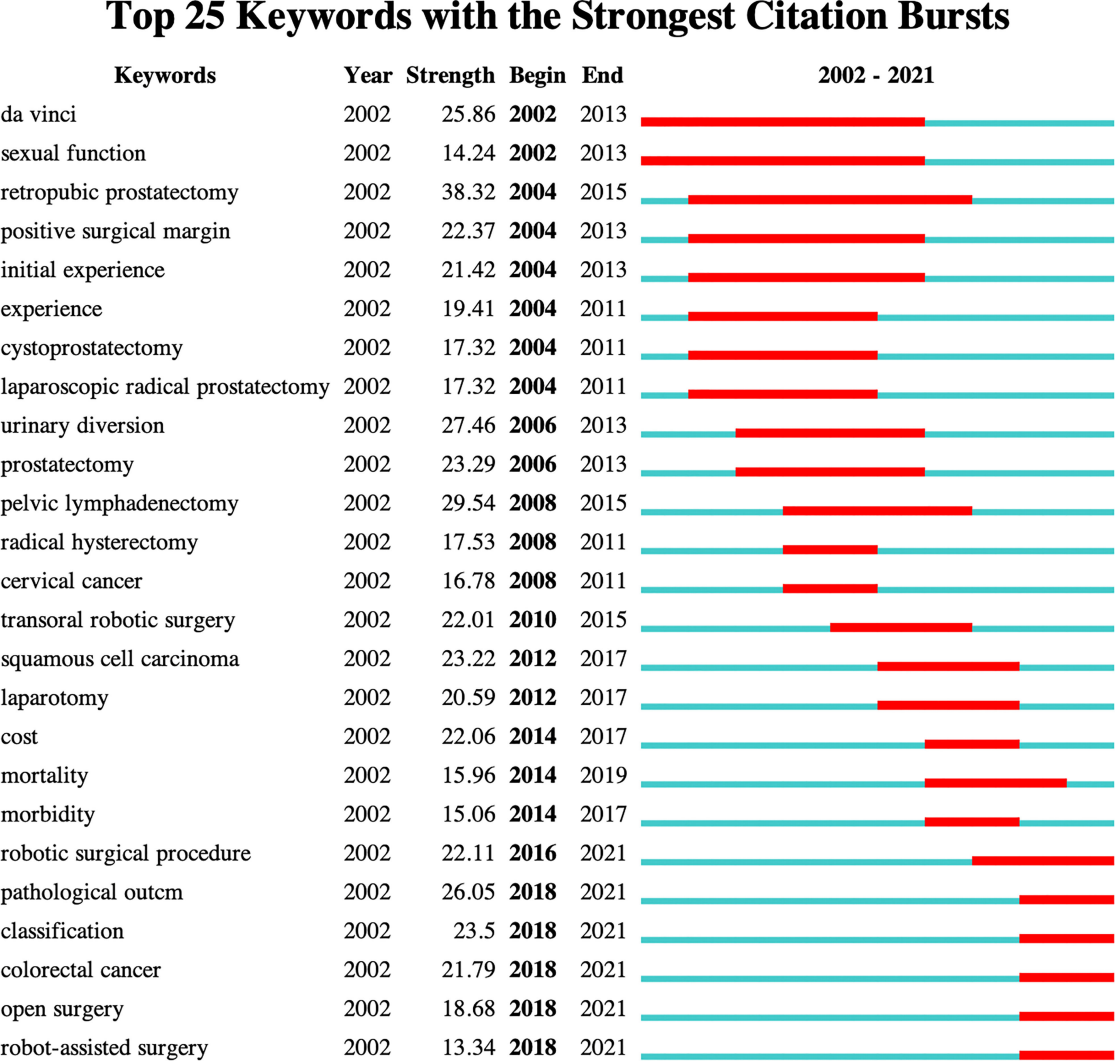
Top 25 keywords with the strongest citation bursts. The blue line indicates the time interval, and the red line indicates the time from the begin to the end of the citation burst.

Keywords are concise summary of the research topic and content in the paper, reflecting the core and essence of the content of the literature, and through the analysis of keywords, to a certain extent, we can grasp the research hotspots and frontiers in the field (61–63). The keywords co-occurrence network knowledge map shows that keywords with a frequency of occurrence of not less than 100 times (**Figure 10**). The top 40 keywords in terms of frequency of occurrence are listed in **Table 8**. The analysis of high frequency keywords shows that robotic surgery, laparoscopic surgery, experience, complications, minimally invasive surgery, resection, learning curve, urologic tumors, colorectal neoplasms, impact, survival, meta-analysis, management, quality of life, risk factors, endometrial cancer, perioperative outcomes, feasibility, short-term outcomes, multicenter, morbidity, laparotomy, and recurrence are the main research topics. The keywords timeline map shows 12 clusters, where keywords of the same cluster are placed on the same horizontal line. The time of occurrence of the keywords is placed at the top of the view, and the time is closer to the right (**Figure 11**). The clustering Q = 0.8882 > 0.3, S = 0.9653 > 0.7, indicating that the structure of the division is significant and the clustering is convincingly efficient (64, 65). The timeline map can clearly obtain the number of keywords in each cluster. The more keywords in the cluster, the more important the cluster field is. At the same time, the time span of keywords in each cluster and the rise, prosperity, and decline of a specific cluster of research can be obtained, to further explore the time characteristics of the research field reflected by the cluster. Analysis of the keyword timeline graph shows that #0 radical prostatectomy, #1 prostate cancer, #3 transoral robotic surgery, #8 rectal cancer, and #10 gastric cancer are the current research hotspots. A “keyword burst” refers to the sudden increase in the frequency of keywords in a short time. By analyzing the burst keywords, we can judge the research hotspots and frontiers in this field (66, 67). The top 25 keywords with the strongest citation bursts were detected by setting the minimum duration of the burst to 3 years (**Figure 12**). Among them, the citation burst of six keywords ended in 2021. Therefore, robotic surgical procedure, pathological outcome, classification, colorectal cancer, open surgery, and robot-assisted surgery reflect the research trends in this field.

**Table 8:** The top 40 keywords in terms of frequency related to robotic surgery in oncology.

Rank	Keyword	Frequency	Rank	Keyword	Frequency
1	robotic surgery	2,901	21	endometrial cancer	301
2	laparoscopic surgery	1,075	22	perioperative outcomes	297
3	experience	773	23	laparoscopic partial nephrectomy	291
4	complications	764	24	feasibility	256
5	minimally invasive surgery	642	25	bladder cancer	253
6	resection	629	26	renal cell carcinoma	248
7	prostate cancer	557	27	short term outcomes	242
8	learning curve	539	28	nephron sparing surgery	217
9	prostatectomy	462	29	multicenter	195
10	rectal cancer	433	30	morbidity	190
11	impact	429	31	radical nephrectomy	182
12	survival	379	32	retropubic prostatectomy	179
13	meta-analysis	376	33	initial experience	176
14	management	372	34	colorectal surgery	173
15	quality of life	347	35	hysterectomy	173
16	total mesorectal excision	344	36	laparotomy	171
17	lymph node dissection	336	37	recurrence	167
18	partial nephrectomy	330	38	low anterior resection	162
19	risk factors	307	39	cystectomy	159
20	lymphadenectomy	305	40	lobectomy	150

### Strengths and limitations

By reviewing the relevant literature, we learned that this scientometric and visual analysis of robotic surgery in oncology research is the first to be reported. We used visualization software to draw a large amount of literature data into scientific knowledge maps, showing the research hotspots and frontiers of robotic oncology surgery in a visual and comprehensive way. However, our study also has some limitations. Firstly, due to the limitations of visual analysis software, it is difficult to merge and analyze data from different databases (PubMed, Scopus, or Embase). Therefore, we only searched the WoSCC database, which may omit some relevant studies. However, it is worth noting that WoS is the most commonly used database for scientometrics research (68–71). And because document type is an important data label for bibliometric analysis, the study reported that the WoS database has more accurate document type assignment than other databases (72). We also imposed certain restrictions on the language and document type in order to make the results of the studies more accurate. Secondly, as relevant literature continues to be published, the academic value of some recently published high-quality studies may be undervalued due to their low citation frequency. Finally, papers published in 2022 were not included in this scientometric and visualization analysis because the database is constantly updated and this year’s dataset is not yet complete. Although there are some limitations, the results of this study can still systematically and comprehensively reflect the research status, hotspots, and trends of robotic surgery in oncology.

## Conclusion

We used the software VOSviewer, CiteSpace, and the Bibliometric Online Analysis Platform to conduct bibliometric and visual analysis of the relevant research on oncology robotic surgery included in the WoSCC database in the past 20 years. It is known that the United States was the country with the most publications, while Yonsei University in South Korea was the most productive institution. The *Journal of Robotic Surgery* and the *Journal of Urology* were the journals with the most publications and citations, respectively. *European Urology* not only ranked in the forefront of publications and citations, but also is an excellent journal with the highest H-index and IF. Mottrie A from Belgium and Ficarra V from Italy were the authors with the highest number of publications and citations, respectively. Menon M from the United States not only ranked in the forefront of publications and citations, but was also the author with the highest average citation per paper and H-index, indicating his significant academic influence in this field. The keywords “robotic surgical procedure”, “laparoscopic surgery”, “prostate cancer”, “colorectal cancer”, “gastric cancer”, “resection”, “complications classification”, “open surgery”, “transoral robotic surgery”, “pathological outcomes”, and “robot-assisted surgery” reflect the research hotspots and trends of oncology robotic surgery.

## Data availability statement

The original contributions presented in the study are included in the article/supplementary material. Further inquiries can be directed to the corresponding author.

## Author contributions

HL, TH, FL and ZH designed the study. HL, FL, JY and ZH contributed to data collection and verification. HL, TH and ZH performed software analysis. HL and ZH drafted the manuscript. TH, FL and JY revised and approved the final version of the manuscript. All authors contributed to the article and approved the submitted version.

## Acknowledgments

Thanks to all study participants for their cooperation.

## Conflict of interest

The authors declare that the research was conducted in the absence of any commercial or financial relationships that could be construed as a potential conflict of interest.

## Publisher’s note

All claims expressed in this article are solely those of the authors and do not necessarily represent those of their affiliated organizations, or those of the publisher, the editors and the reviewers. Any product that may be evaluated in this article, or claim that may be made by its manufacturer, is not guaranteed or endorsed by the publisher.
